# Synthesis of salt-stable fluorescent nanoparticles (quantum dots) by polyextremophile halophilic bacteria

**DOI:** 10.1038/s41598-018-38330-8

**Published:** 2019-02-13

**Authors:** N. Bruna, B. Collao, A. Tello, P. Caravantes, N. Díaz-Silva, J. P. Monrás, N. Órdenes-Aenishanslins, M. Flores, R. Espinoza-Gonzalez, D. Bravo, J. M. Pérez-Donoso

**Affiliations:** 1 BioNanotechnology and Microbiology Lab, Center for Bioinformatics and Integrative Biology (CBIB), Universidad Andres Bello, Santiago, Chile; 2 Laboratorio de Nanotecnología, Recursos Naturales y Sistemas Complejos, Facultad de Ciencias Naturales, Departamento de Química y Biología, Universidad de Atacama, Copiapó, Chile; 3 Departamento de Ingeniería Química, Biotecnología y Materiales, Facultad de Ciencias Físicas y Matemáticas, Universidad de Chile, Santiago, Chile; 4 Laboratorio de Microbiología Oral, Facultad de Odontología, Universidad de Chile, Santiago, Chile

## Abstract

Here we report the biological synthesis of CdS fluorescent nanoparticles (Quantum Dots, QDs) by polyextremophile halophilic bacteria isolated from Atacama Salt Flat (Chile), Uyuni Salt Flat (Bolivia) and the Dead Sea (Israel). In particular, a *Halobacillus* sp. DS2, a strain presenting high resistance to NaCl (3–22%), acidic pH (1–4) and cadmium (CdCl_2_ MIC: 1,375 mM) was used for QDs biosynthesis studies. *Halobacillus* sp. synthesize CdS QDs in presence of high NaCl concentrations in a process related with their capacity to generate S^2−^ in these conditions. Biosynthesized QDs were purified, characterized and their stability at different NaCl concentrations determined. Hexagonal nanoparticles with highly defined structures (hexagonal phase), monodisperse size distribution (2–5 nm) and composed by CdS, NaCl and cysteine were determined by TEM, EDX, HRXPS and FTIR. In addition, QDs biosynthesized by *Halobacillus* sp. DS2 displayed increased tolerance to NaCl when compared to QDs produced chemically or biosynthesized by non-halophilic bacteria. This is the first report of biological synthesis of salt-stable QDs and confirms the potential of using extremophile microorganisms to produce novel nanoparticles. Obtained results constitute a new alternative to improve QDs properties, and as consequence, to increase their industrial and biomedical applications.

## Introduction

Semiconductor nanocrystals or Quantum Dots (QDs) exhibit unique optical and electronic properties with fluorescence emission wavelengths depending on nanoparticle (NPs) size and composition^1–3^. Nowadays, the interest in studying QDs has increased due to their numerous applications in photovoltaics, optoelectronics, transistors, oil exploration, biomedicine and imaging, among others^3–7^.

To date, QDs are mainly obtained through chemical synthesis; however, most of these methods involve the use of toxic reagents, high temperatures and anaerobic conditions. In addition, most QDs synthesized by chemical methods present low biocompatibility and poor stability at high osmolarity conditions, thus affecting their potential applications^5,8–11^. In this context, there is a growing interest in the generation of green and eco friendly methods for QDs production to be used in different technological applications.

During the last years, the industrial and scientific interest for developing eco-friendly and sustainable methods to synthesize CdS, CdSe and CdTe QDs has grown. The addition to chemical synthesis procedures of bidentate thiols [e.g. dithiothreitol (DTT), mercaptosuccinic acid (MSA), mercaptopropionic acid (MPA)] and ligands with different functional groups (amino, hydroxyl, carboxylic acid, among others) has been used to improve NPs biocompatibility and stability^12^. Nevertheless, these methods produce NPs that still display low biocompatibility, sensitivity to pH and high ionic strength, as well as elevated production costs^11,13–15^.

Several chemical methods based on the use of biological reagents and mild conditions have been developed during the last years^16–19^. These methods, denominated biomimetic, have allowed the production of nanomaterials in a simpler and eco-friendly way. In addition, biomimetic procedures have contributed to improve the properties of NPs, being the increase on biocompatibility one of the most significant improvements since it has a direct impact on the range of nanoparticles applications^14,15^.

Most biomimetic methods described to date involve the use of mild temperatures (90 °C), aerobic conditions, and biological molecules, such as biological thiols^17–19^. Other biological components such as peptides, nucleotides, fusion proteins and phosphorylated molecules have also been used for the chemical synthesis of QDs^14,16,18–21^.

In this context, biological synthesis of nanoparticles has emerged as a sustainable and green alternative to classical production methods. Several protocols reporting the production of NPs using cell extracts or living cells have been developed during the last decade. These biologically produced nanoparticles display high stability, water solubility, biocompatibility, low costs and high production rates, among others^22–24^. From these protocols, the biosynthesis of NPs using microorganisms has been implemented as a cost effective and eco-friendly alternative.

Several microorganisms, including bacteria and fungi, are used to biosynthesize cadmium-based QDs^24–28^. Most biosynthesis processes reported to date involve different thiolated biomolecules as relevant precursors of the biosynthetic process^29–31^. Moreover, the importance of biological thiols such as glutathione and H_2_S has been recently showed^26,27,32,33^. All these reports relate the biosynthesis of QDs with the presence of biological thiols promoting the intra- and extracellular generation of QDs. Recently, our group determined the importance of phosphorylated biomolecules in the biological synthesis of CdS QDs. Phosphate groups present in biologically relevant molecules such as nucleotides and metabolic sugars, contribute to the generation of the nanocrystal and constitute the external layer of the QDs^16^. Despite these advances, the molecular mechanism involved in QDs biosynthesis is still unknown^16,23,25,34^.

With the aim of finding new methods to biosynthesize QDs with improved properties, our group has focused on using bacteria inhabiting extreme environments (extremophiles) for QDs production. Accordingly, we reported the biological synthesis of QDs at low temperatures using novel psychrotolerant bacterial strains isolated from Antarctica^26,27^. In addition, our group recently reported the production of fluorescent Cd-nanoparticles by acidophilic bacteria used in biomining operations^35,36^. Bacteria of the *Acidithiobacillus* gender (*A. ferrooxidans*, *A. thiooxidans* and *A. caldus*) were used to biosynthesize Cd-QDs under acidic conditions (pH 3,0). Interestingly, QDs produced by acidophilic bacteria were highly stable at low pHs, a characteristic not observed in QDs produced by chemical methods or biosynthesized by the mesophilic bacteria *Escherichia coli*^35^. To date, the use of other extremophile microorganisms to produce QDs with novel properties has not been explored.

Most halophilic microorganisms inhabit soil or liquid environments characterized by the presence of high salt concentrations^37–39^. Halophilic prokaryotes, belonging to Bacteria or Archaea domains, can adapt to a wide range of osmolarities, some of them living near NaCl saturation^37^. Salt-tolerant bacteria have been isolated from a wide range of environments at all latitudes^40–42^. To date, most halophilic microorganisms have been isolated with the purpose of obtaining new biomolecules of industrial interest like enzymes, exopolysaccharides and phytohormones, among many others^43^. However, their use in the biosynthesis of fluorescent nanoparticles has not been explored.

Salt stable nanomaterials are a useful alternative for different applications, particularly for those involving NPs transport in presence of ionic solutions such as real time sensing in oil explorationand bioimaging^5,44^. The injection of transportable fluorescent nanoparticles would allow spatially resolved measurements of temperature, pressure, rock porosity and permeability of an oil reservoir^5^. In addition, discovering and characterizing the biomolecules involved in fluorescence salt-stability of biosynthesized nanocrystals would allow the development of new chemical methods to produce NPs with improved properties.

The objective of the present work was to determine if halophilic microorganisms isolated from extreme environments with high salt concentrations (Atacama Desert, Uyuni Salt Flat and Dead Sea) can biosynthesize Cd-QDs under high osmolarity conditions. In addition, we characterized the QDs produced by halophilic bacterium and we tested the ability of QDs to retain their fluorescence intensity in presence of elevated salt concentrations (fluorescence stability), a condition affecting most QDs described to date.

## Results

### Isolation and characterization of halophilic bacteria

15 bacterial isolates able to grow in Luria Bertani (LB) medium supplemented with NaCl 8 or 15% at different temperatures (28 to 37 °C) were obtained from Atacama Salt Flat (ASF), Uyuni Salt Flat (USF) and Dead Sea (DS) samples, as described in methods.

The ability of all isolates to grow at different NaCl concentrations was determined (Table 1). Obtained results indicate that bacterial isolates are able to grow in a wide range of NaCl concentrations (1 to 22%). All isolates were also capable to grow at 28 and 37 °C. Based on these results, these microorganisms can be classified as halophiles. Therefore, they present a unique opportunity to study the process of QDs biosynthesis given the extreme conditions in which they can survive.

**Table 1:** Cadmium and NaCl tolerance of halophilic isolates.

Isolate	% NaCl Range	CdCl_2_ MIC (mM)
ASF1	2–15	1.375
ASF2	2–15	0.66
ASF3	2–15	1.375
ASF4	2–15	0.66
ASF5	2–15	1.375
UYSF1	2–22	1.375
UYSF2	2–22	0.66
UYSF3	2–22	0.66
UYSF4	1–25	1.375
UYSF5	1–25	1.375
DS1	4–25	0.66
DS2	3–22	1.375
DS3	3–22	0.66
DS4	3–22	0.66
DS5	3–22	1.375

Cadmium MICs and ranges of NaCl concentrations allowing bacterial growth are shown.

Most CdS QDs biosynthetic methods described to date involve the use of cadmium salts such as CdCl_2_. Based on this, CdCl_2_ resistance of isolated halophiles was determined. Minimal inhibitory concentration (MIC) values ranging from 0.66 to 1.375 mM (121 to 251.15 μg/mL) were obtained (Table 1). These values are similar to other strains widely used for QDs biosynthesis belonging to different species such as *P. aeruginosa* PAO1 (0.55 mM or 100 μg/mL) and *E. coli* (1.265 mM or 230 μg/mL)^27^. Obtained results indicate that eight halophilic isolates are resistant to cadmium (1.375 mM).

### Biosynthesis of CdS QDs by halophilic bacteria

**Figure 1:**
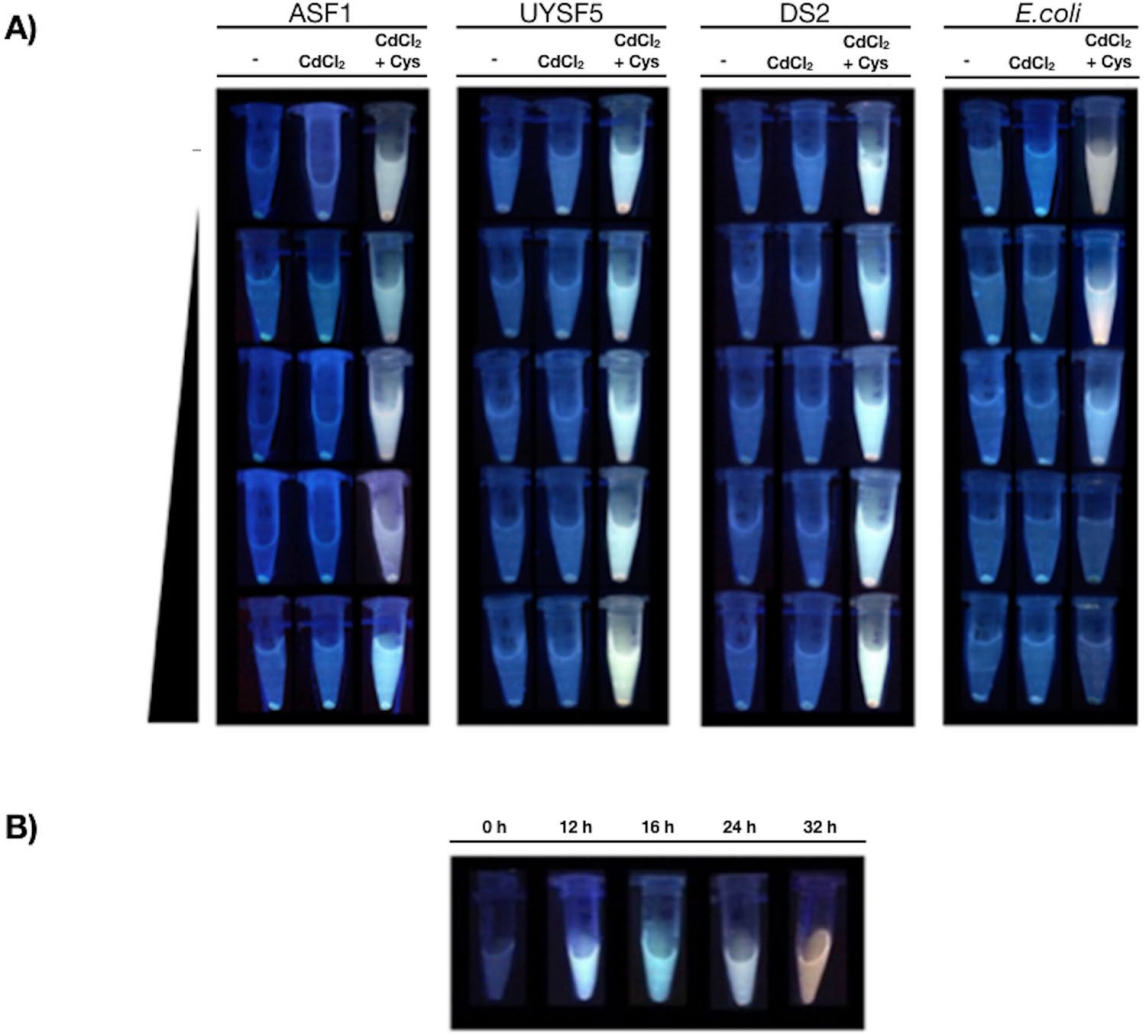
Biosynthesis of QDs in presence of different NaCl concentrations by halophilic isolates. (**A**) The biosynthesis of QDs was evaluated in the halophilic isolates ASF1, UYSF5, DS2 and the control bacteria *E. coli*. The effect of NaCl (0, 2, 4, 6 and 8%) on QDs biosynthesis in presence or absence of cysteine (2 mM) was evaluated. (**B**) DS2 was exposed to QDs-biosynthesis conditions and QDs fluorescence after UV exposure was evaluated at different times of biosynthesis (0, 12, 16, 24 and 32 h).

Based on the characteristics of the halophilic strains isolated, we decided to evaluate CdS QDs biosynthesis in culture media supplemented with NaCl 8%. We mainly focus in extracellular biosynthesis since this method facilitates NPs purification and favors future applications. The capacity of all obtained isolates to biosynthesize QDs was evaluated following the protocol described before^4,16,26,27,36^. Only three cadmium-resistant isolates were able to biosynthesize cadmium-based QDs in the presence of NaCl up to 8%, as determined by fluorescence of cell supernatants (Fig. 1A). The strains with the capacity to biosynthesize QDs were ASF1, UYSF5 and DS2, isolated from Atacama Salt Flat, Uyuni Salt Flat and Dead Sea, respectively. The three isolates capable of biosynthesizing QDs were those that also displayed the highest resistance to Cd (1.375 mM) and NaCl (15% or more) (Table 1) among all isolates. *E. coli* was used as control, since it has been widely used to study biosynthesis of fluorescent NPs^16,25,32^. As expected, *E. coli* was unable to biosynthesize QDs in presence of NaCl 6% or higher.

The extremophile strain DS2 was selected for the following experiments since it was able to synthesize QDs, grew at high NaCl concentrations (3–22%) and exhibited resistance to CdCl_2_ (1.375 mM). In addition, this isolate was able to grow in a wide range of pHs (pH 1–9). The 16S rRNA gene of DS2 was amplified and sequenced to determine the identity of this strain. The obtained sequence showed that DS2 display high identity with *Halobacillus* sp. (sequence identity: 96–98%), a halophilic spore forming bacterium previously isolated from salt environments^45^.

To confirm that the fluorescence observed during biosynthesis corresponds to QDs, time dependent changes in the fluorescence of *Halobacillus* DS2 cells exposed to biosynthetic conditions were evaluated (cysteine 1 mM, NaCl 8% and CdCl_2_ 0.33 mM). Supernatant aliquots were collected at several times and analyzed to evaluate extracellular synthesis of NPs. Fluorescence emission colors changed at different times, moving from blue to red after 32 h (Fig. 1B). This behaviour is characteristic of QDs formation and it has been related to increasing nanocrystal size, which changes the band gap of nanoparticles and their spectroscopic properties^16,26,27,32,35^. In this context, obtained results are very novel since QDs biosynthesis in the presence of high concentrations of NaCl or using halophilic strains has not been reported to date.

### H_2_S production at high osmolarity is required for QDs biosynthesis

**Figure 2:**
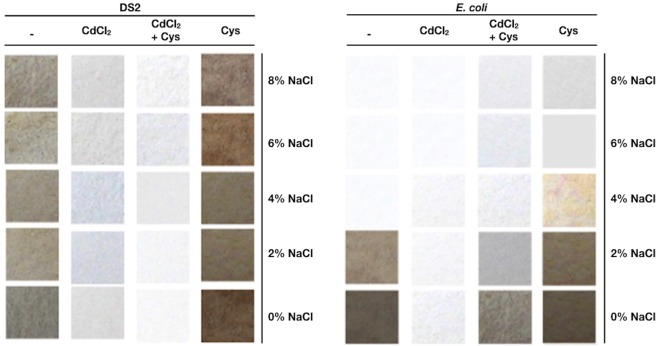
Effect of NaCl on *Halobacillus* sp. DS2 sulfide production. The production of sulfide by *Halobacillus* DS2 and *E. coli* in presence of different NaCl concentrations was determined using papers soaked with lead acetate (Shatalin *et al*., 2011). (−) Cells in LB. (CdCl_2_) cells in LB supplemented with CdCl_2_. (CdCl_2_ + Cys) Cells in LB supplemented with cysteine and CdCl_2_. (Cys) Cells in LB supplemented with cysteine.

Hydrogen sulfide has been described as a promoter molecule for the biosynthesis process of sulfur-containing nanoparticles^16,26,27,35^. Based on this, H_2_S production was assessed in LB media supplemented with several NaCl concentrations. Since bacterial sulfide production has been associated with the activity of cysteine desulfhydrase^46^, we determined sulfide generation in the presence of cysteine (substrate of cysteine desulfhydrase enzyme). The generation of black precipitates on lead acetate papers exposed to cultures growing in LB media was positive for *E. coli* at 0 and 2% NaCl, and at all NaCl concentrations tested for the *Halobacillus* DS2 isolate (Fig. 2). When cysteine was added to bacterial cultures, an increase in sulfide generation was observed at all NaCl concentrations. In the case of *E. coli*, cysteine allowed the production of sulfide at 0 and 2% NaCl, and scarce amounts were detected at higher NaCl concentrations (Fig. 2).

H_2_S production under conditions of Cd-based QDs biosynthesis was also evaluated. A decrease in sulfide levels produced by *Halobacillus* sp. DS2 and *E. coli* cells was observed at all NaCl concentrations tested, a result that has been associated with the extracellular generation of CdS nanocrystals as consequence of the interaction of sulfide and the metal ion^27,47^. In summary, H_2_S assays indicated that the *Halobacillus* sp. DS2 isolate is capable of synthesizing sulfide even in presence of high salt concentrations, and also that the generation of this compound is associated with CdS generation in culture supernatants.

### Spectroscopic characterization of biosynthesized QDs

**Figure 3:**
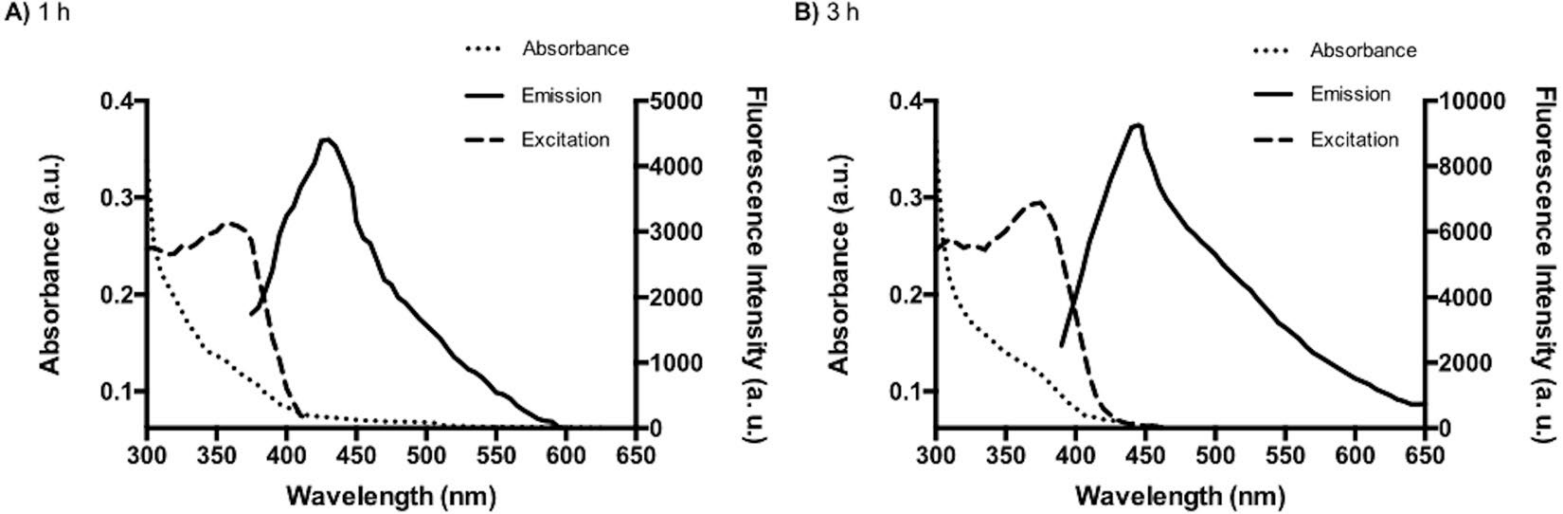
Absorbance, fluorescence and excitation spectra of QDs produced by *Halobacillus* sp. DS2. (**A**) Absorbance, emission and excitation spectra of QDs biosynthesized at 1 h. The emission was registered at 450 nm for excitation spectra. (**B**) Absorbance, fluorescence emission and fluorescence excitation spectra of QDs biosynthesized at 3 h. The emission was registered at 450 nm for excitation spectra.

Fluorescent nanoparticles produced by *Halobacillus* sp. DS2 after 1 and 3 h synthesis were purified from cells following the protocol previously described by Ulloa *et al*., 2016. Purified nanoparticles were used for all characterization experiments. As shown in Fig. 3A, the absorbance profile of purified QDs presents a small peak at 360 nm characteristic of biosynthesized QDs^32^. Based on these spectra, we calculated the size of the QDs using the Henglein’s empirical model, which relates the diameter of CdS QDs to the absorption UV-vis spectra^48–50^. QDs size of 4.4 and 5.0 nm were predicted for NPs biosinthesized at 1 and 3 h, respectively.

Also, the fluorescence profile of purified nanoparticles produced by *Halobacillus* sp. DS2 after 1 or 3 h displayed emission peaks between 420–500 and 440–530 nm, respectively (Fig. 3B). These fluorescence spectra are characteristic of CdS QDs and the differences in emission observed between both QDs are related to differences in the size of the nanocrystal^16,17,19,28^. Interestingly, a high quantum yield (23.53%) was determined in QDs produced by *Halobacillus* sp. DS2. This is a desirable property for bioimaging applications that is not commonly observed on biosynthesized QDs^16,26,27,32,35^. In addition, the excitation spectra of QDs biosynthesized at 1 and 3 h were determined (Fig. 3C). Excitation peaks at 360 and 365 nm, characteristic of CdS QDs, were observed for QDs produced *by Halobacillus* sp. DS2 after 1 and 3 h synthesis (emission fluorescence was measured at 450 and 500 nm, respectively)^32,51,52^. In QDs, the fluorescence spectrum is independent of the excitation wavelength due to dissipation of the excitation energy during the excited state (Kasha rules)^53^_._

### QDs produced by *Halobacillus* sp. DS2 are stable at high NaCl concentrations

**Figure 4:**
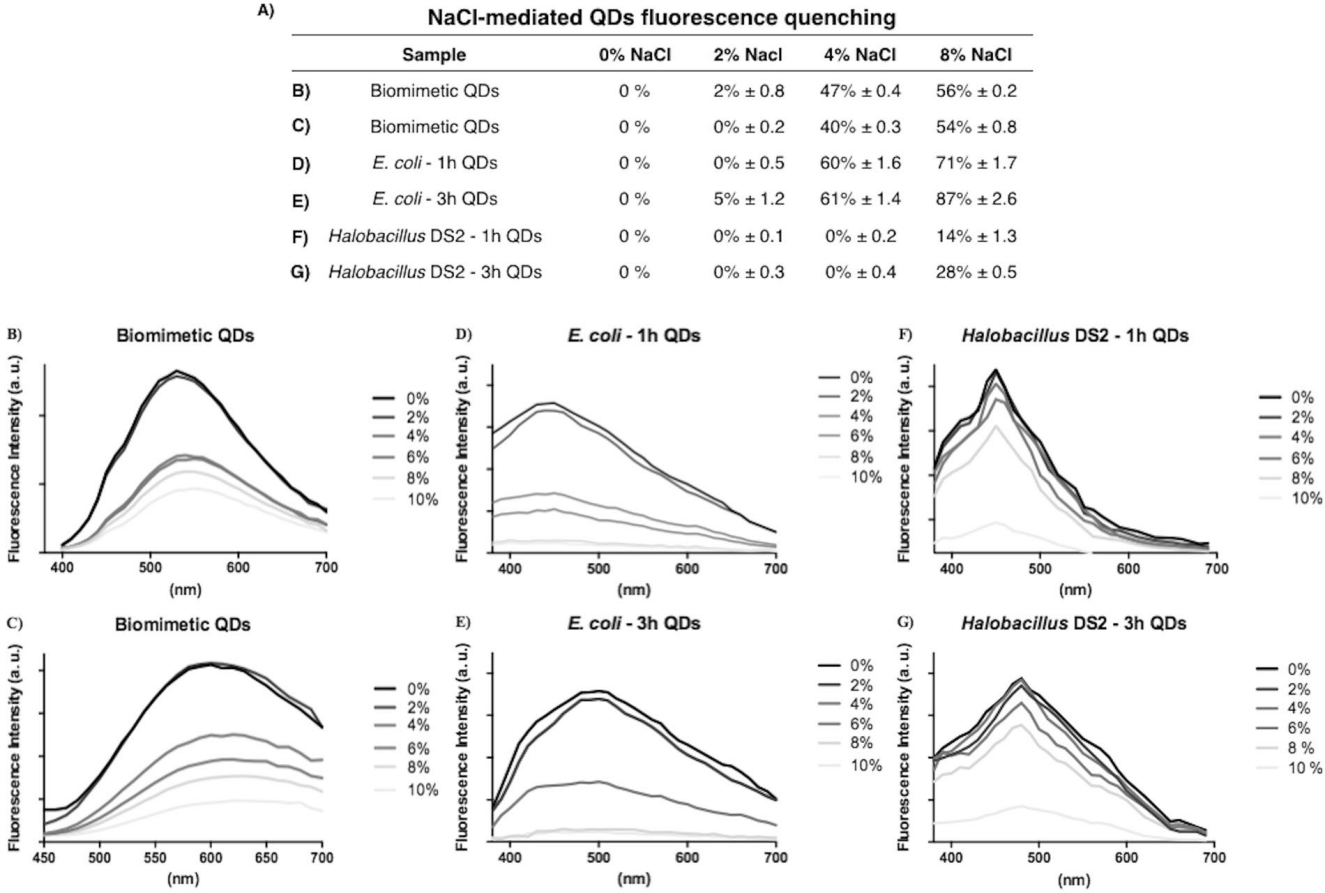
CdS QDs biosynthesized by *Halobacillus* sp. DS2 are stable at high NaCl concentrations. (**A**) Effect of different NaCl concentrations on the fluorescence emission intensity (percentage of quenching) and spectra of green and red QDs synthesized by a chemical method (**B,C**), *E. coli* (**D,E**) or *Halobacillus* sp. DS2 (**F,G**).

Considering that *Halobacillus* sp. DS2 is highly resistant to NaCl, and also that this isolate is capable of synthesizing QDs in the presence of different salt concentrations, we decided to evaluate the stability of biosynthesized QDs at high NaCl concentrations. QDs produced by a chemical method and QDs biosynthesized by *E. coli* were also analyzed as controls. QDs were incubated 30 min with different NaCl concentrations and the effect on QDs-associated fluorescence was determined. As shown in Fig. 4, QDs biosynthesized by the *Halobacillus* sp. DS2 maintain their fluorescence properties even after exposure to 4% NaCl; no changes on fluorescence intensity were observed at 2 and 4% NaCl (0% fluorescence quenching). In contrast, a quenching in fluorescence higher than 40% was observed when biomimetic QDs or those biosynthesized by *E. coli* were exposed to 4% NaCl. The exposure to 8% NaCl produces a fluorescence quenching of 14 and 28% on QDs biosynthesized during 1 and 3 h by *Halobacillus* sp. DS2, respectively. Interestingly, a fluorescence quenching higher than 70% was observed in QDs biosynthesized by *E. coli* under the same conditions. These results confirm that QDs produced by *Halobacillus* sp. DS2 are stable at high salt concentrations, a unique characteristic probably related with changes in composition or structure of the nanocrystal produced by the halophilic bacterial isolate.

### Characterization of QDs

**Figure 5:**
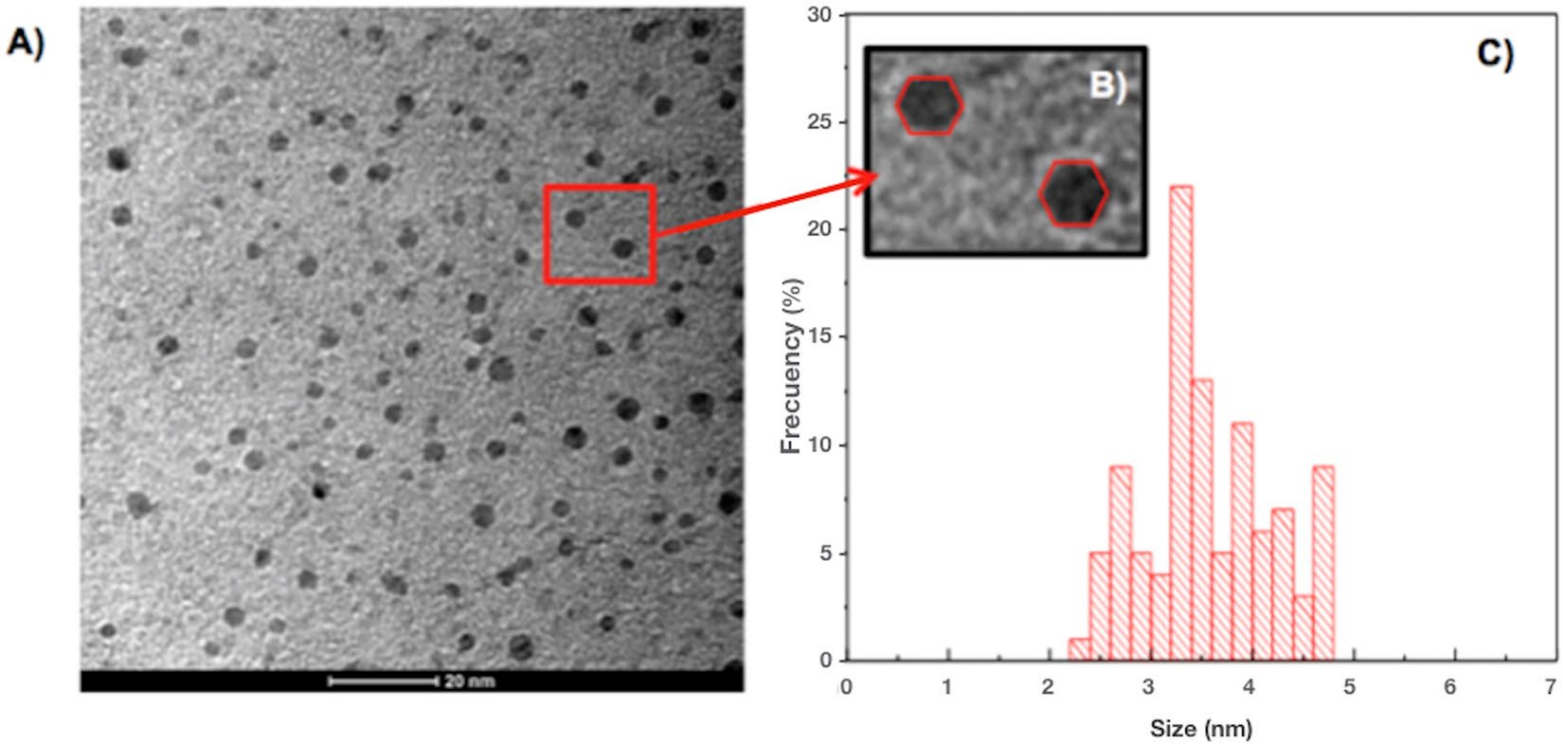
TEM analysis of purified CdS QDs biosynthesized by *Halobacillus* sp. DS2. (**A**) Representative TEM image of biosynthesized CdS QDs. (**B**) Digital zoom of the TEM image of QDs. (**C**) Frequency size histogram of biosynthesized QDs.

Extracellular CdS-QDs produced after 1 h synthesis were purified and characterized by TEM (Fig. 5A). The electron micrograph shows that NPs are uniformly distributed in an amorphous matrix of low crystallinity. A digital zoom of the TEM image shows that these QDs are regular polyhedra (Fig. 5B), similar to models of hexagonal NPs^54^. Figure 5C show the size frequency histogram and the average size of biosynthesized NPs (3.56 nm).

**Figure 6:**
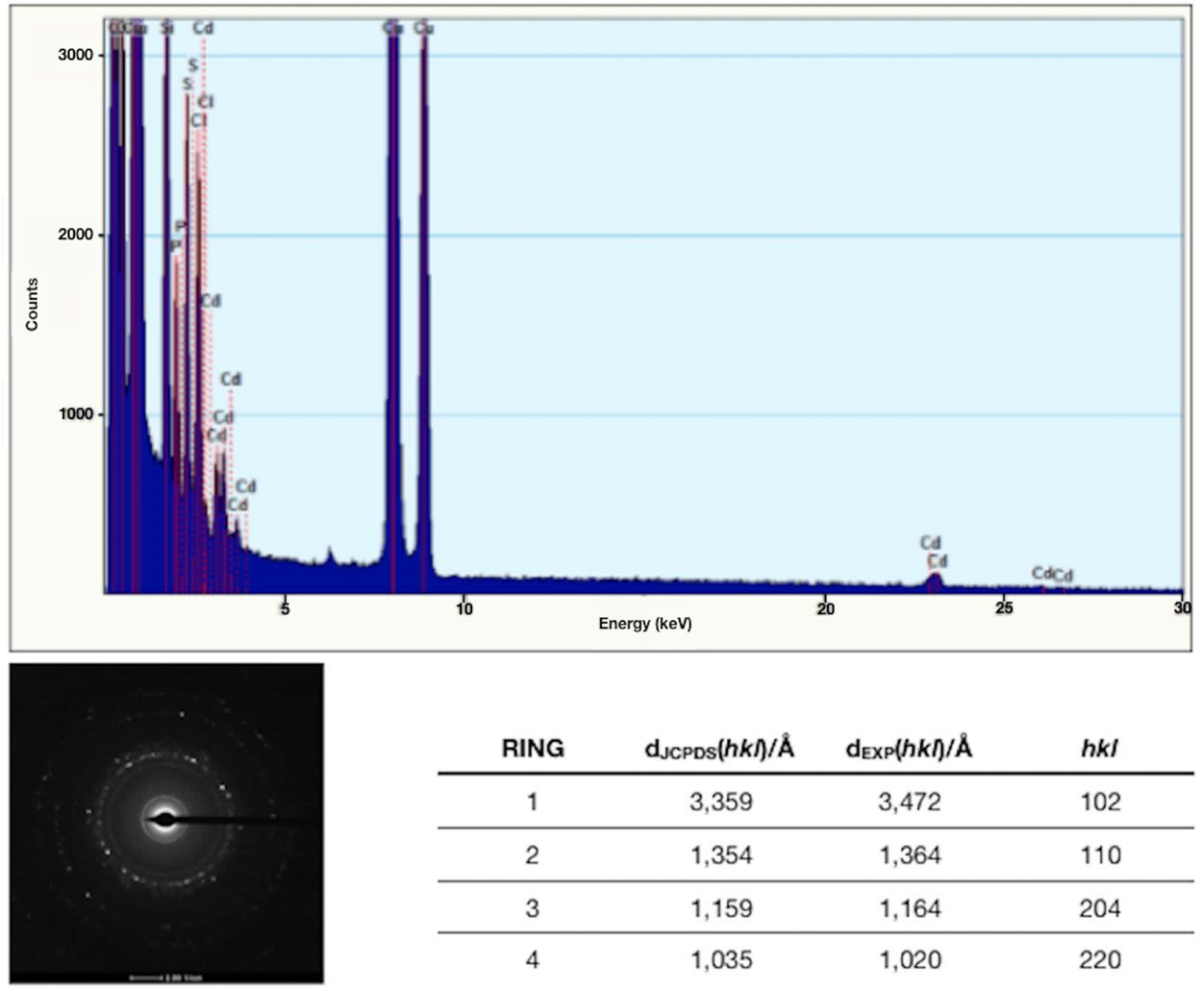
EDS characterization of purified CdS QDs biosynthesized by *Halobacillus* sp. DS2. (**A**) EDS of biosynthesized QDs. (**B**) Electron diffraction pattern of QDs. (**C**) Interplanar spaces of biosynthesized QDs and those corresponding to hexagonal CdS of the JCPDS 41-1049 file.

Energy dispersive X-ray spectroscopy (EDS) analysis showed that QDs are composed of cadmium and sulfide, while the amorphous matrix contains carbon, oxygen, phosphorus and chlorine (Fig. 6A,B). Most of these elements are characteristic of biomolecules bound to the nanocrystal. By electron diffraction we obtained patterns with the interplanar distances of biosynthesized QDs (Fig. 6C). The SAED pattern shows that biosynthesized NPs have a crystalline structure with interplanar distances of d_1_ = 0.245 nm, d_2_ = 0.207 nm, d_3_ = 0.123 and d_4_ = 0,104 nm. This is in agreement with previously reported data indicating that these interplanar distances correspond to the structure of CdS nanocrystals, specifically polycrystalline wurtzite (hexagonal) nanoparticles^55^. Four diffraction rings consistent with spacings corresponding to a hexagonal phase of cadmium sulfide were observed in the NPs produced by *Halobacillus* sp. DS2.

**Figure 7:**
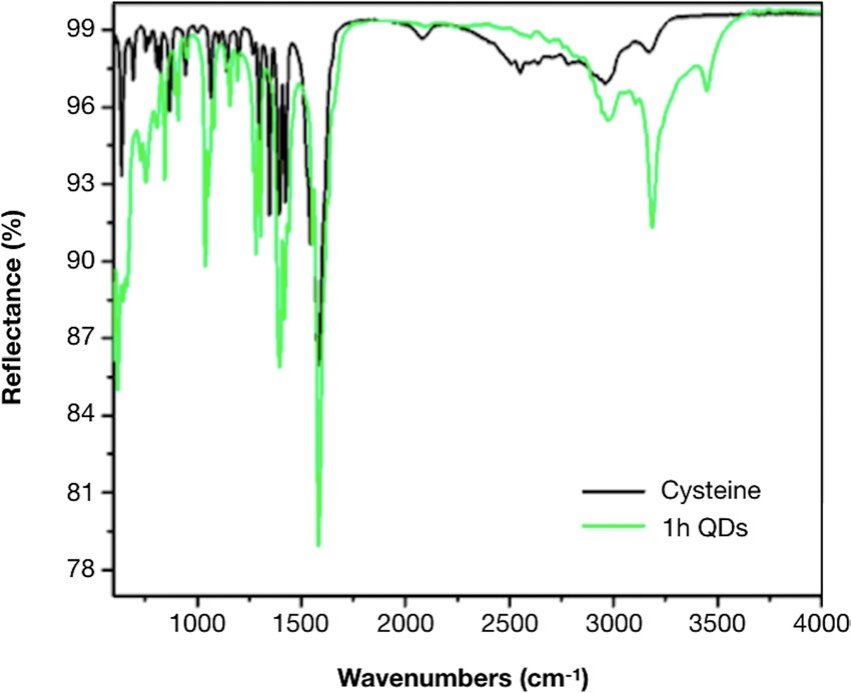
FTIR analysis of CdS QDs biosynthesized by *Halobacillus* sp. DS2. FTIR spectrum of cysteine (**A**) and biosynthesized QDs (**B**).

The FTIR spectrum for CdS-QDs and cysteine are shown in Fig. 7. In both spectra, a peak close to 1575 cm^−1^ corresponding to carboxylate group is observed. However, for both spectra, this peak is shifted to lower wavelengths, between 1570 and 1690 cm^−1^. This shift can be due to the formation of a amino-acid Zwitterion^55^. In that case, the carboxilate group presents a resonance effect in which both C-O bonds share the electron charge of the double bond of C=O, then the bond multiplicity is lower and the vibration frequency changes. The characteristic band for N–H stretching modes is observed at 3346 and 3250 cm^−1^ indicating the presence of an NH_2_ group. Also, the S-H stretching mode is clearly seen at 2550 cm^−1^, as expected for cysteine. Interestingly, this band is not present in the spectrum of CdS–QDs. This is likely the result of covalent bond formation between the thiol and Cd at the surface of CdS-QDs.

**Figure 8:**
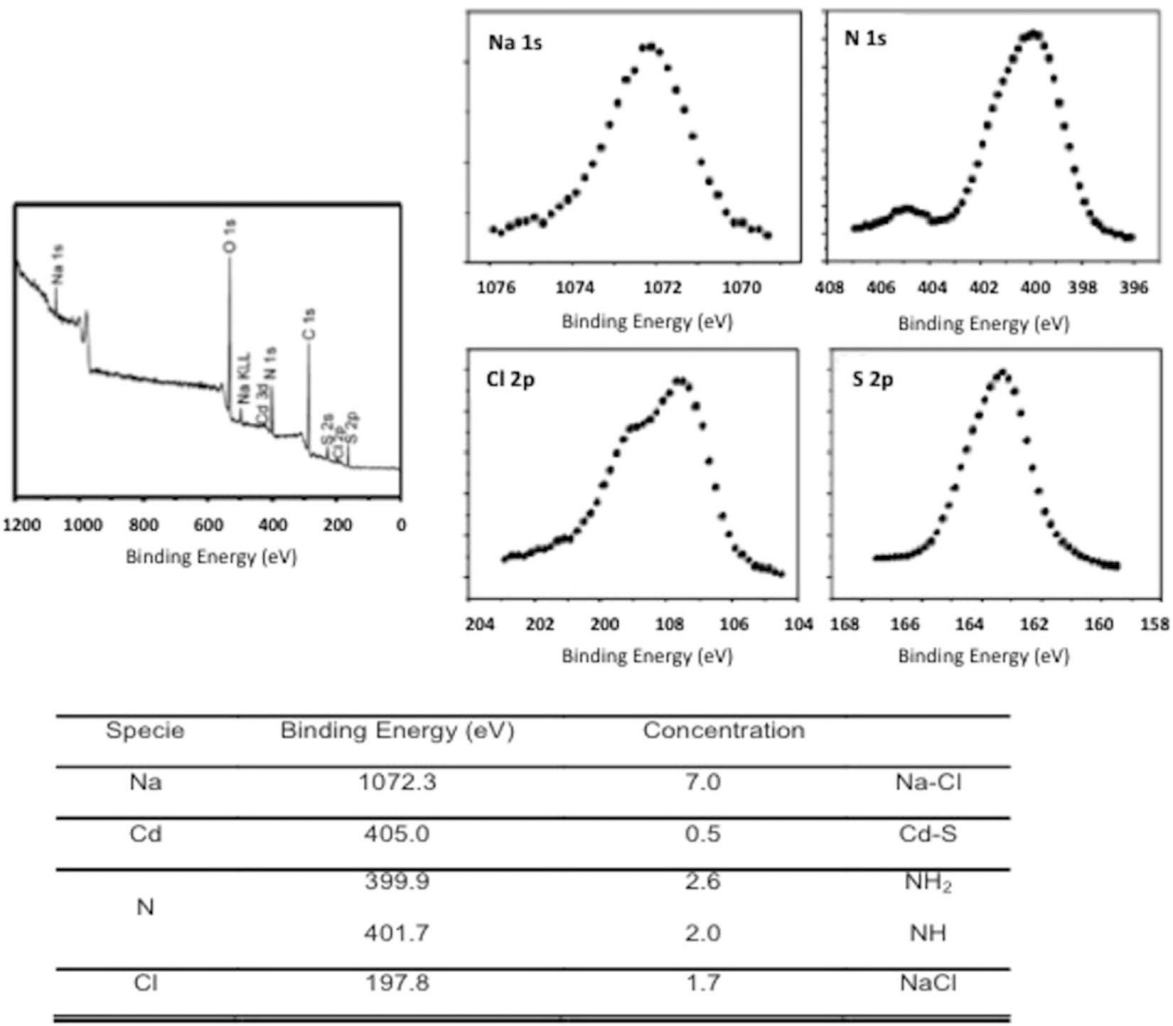
HRXPS characterization of CdS-QDs biosynthesized by *Halobacillus* sp. DS2.

An HRXPS characterization was performed in biosynthesized QDs. The full spectrum shows the presence of several chemical species (Fig. 8). The identification of the binding energies is linked with the different molecules and the NPs in the sample. Sodium and chlorine, Na1s and Cl2p respectively, are mainly attributed to NaCl. The fit of the sulphur S2p revealed two components that can be attributed to CdS NPs and cysteine molecules. Finally, the nitrogen N1s shows two peaks corresponding to NH_2_ and NH present in cysteine.

## Discussion

Fifteen salt-resistant bacteria were isolated from three different extreme environments characterized by the presence of high salt concentrations. Growth of bacterial isolates at different salt concentrations and pHs were evaluated, being most of them able to grow in a wide range of pHs (particularly acidic) and salt concentrations (3–25%).

Several reports have described that extreme halophiles belonging to different phylogenetic phyla such as Actinobacteria, Bacteroidetes and Alpha-proteobacteria exhibit unique physiological and nutritional properties^56^. The halophilic isolates studied here grow in medium supplemented with up to 15% NaCl and given that they are able to grow optimally at NaCl concentrations higher than 10% (1.7 M) they can be categorized as extreme halophiles^57^. Other halophiles are able to grow optimally at lower salt concentrations, like those present in the sea (3.5%, ~0.6 M)^56,58^.

The extremophile strain DS2 was able to synthesize QDs, grew at high NaCl concentrations and exhibited resistance to CdCl_2_ (1.375 mM). The 16S rRNA gene analysis revealed that the DS2 isolate displays high identity with *Halobacillus* sp. Several *Halobacillus* isolates have been described to date, most of them displaying high tolerance to NaCl (1–25%) and heavy metals^45^. Additionally, the capacity to grow at pH ranges between 5–8 was reported on one isolate obtained from a salt mine in Pakistan^59^.

The *Halobacillus* sp. DS2 isolate was also able to grow in a wide range of pHs (pH 1–9). Based on these results, the *Halobacillus* sp. DS2 isolate can be classified as polyextremophile (acidophilic extreme halophile), since it can grow at acidic pHs (pH 1–6), high concentrations of NaCl and can tolerate elevated concentrations of the heavy metal cadmium^60^.

Three halophilic isolates were able to synthesize CdS quantum dots in presence of high NaCl concentrations (6–8%); a result somehow surprising since the biosynthesis of QDs under high salinity concentrations has not been reported before. *E. coli* (a classical model of QDs biosynthesis) is unable to biosynthesize QDs in presence of NaCl 4% or higher (Fig. 1). This result is related at least in part to the inability of *E. coli* to produce H_2_S in presence of high NaCl concentrations as determined in Fig. 2.

A few reports have shown the effect of Na^+^ over the cellular production of H_2_S. In cyanobacteria and plants it has been described that sodium toxicity is due in part to the ability of this ion to interact with the 3-phosphoadenosine-5-phosphatase (PAPase) and to inhibit their activity removing the toxic intermediary of S-assimilation, 3-phosphoadenosine-5-phosphate (PAP)^61,62^. PAP accumulation in *E. coli* could be related to a reduction of H_2_S production through the enzyme PAPs reductase (encoded by *cysH* in *E. coli*) that catalyzes the production of sulfite. PAP accumulation generates a product inhibition of PAP reductase, thus decreasing the formation of sulfite, a substrate required for the formation of H_2_S by the sulfite reductase enzyme (encoded by *cysJI* and *cysG* in *E. coli*).

In previous reports it has been described the existence of sodium-tolerant PAPases in halophilic microorganisms, such as the case of *halA* from the cyanobacterium *Arthospira platensis*^62^. Based on the results obtained in cultures amended with cysteine, all isolates analyzed have desulfhydrase enzymes (EC 4.4.1.15), a large family of enzymes that catalyze the conversion of cysteine in sulfide, ammonium and pyruvate, and vice versa. The effect of NaCl on the cysteine desulfhydrase of the halophilic strain *Halobacterium salinarum* has been previously studied^63^. It was shown that the purified desulfhydrase enzyme from *H. salinarum* has a lower sensitivity to NaCl than the homologous enzyme from *E. coli*^63^.

QDs produced by *Halobacillus* sp. DS2 presented high fluorescence stability under osmotic stress conditions, particularly when compared with chemical QDs and biological QDs synthesized by *E. coli* (Fig. 2). To date, the biological production of QDs with novel or improved properties has been scarcely studied. Our group recently reported the biosynthesis of QDs with increased tolerance to acidic conditions by using acidophilic extremophile bacteria^35,36^. We also determined that metal resistant psychrotolerant bacteria isolated from Antarctica could synthesize Cd-QDs at low temperatures^26,27^. Also, the use of Antarctic bacteria to biosynthesize CdS QDs in presence of other toxic metal contaminants (arsenic) was recently reported as a new alternative for bioremediation^64^. The cellular processes involved in the synthesis of NPs in all these bacteria isolated from extreme environments have not been determined, however, the importance of sulfide on the biosynthesis process has been established^26,27,35,64^.

The novel properties of QDs produced by extremophile bacteria are most probably related to the generation of nanocrystals with novel compositions and/or structures, probably as consequence of the presence/activity of cellular biomolecules. This last point has been scarcely studied in QDs produced through biological approaches. Because of this, in the present work we performed a detailed chemical and structural characterization of the CdS QDs produced by halophilic bacteria.

The electron microscopy image shows that CdS QDs have an average size of 3.6 ± 0.8 nm with low size polydispersity. Hexagonal NPs structures were observed in QDs biosynthesized by *Halobacillus* sp. DS2 (Fig. 4B). QDs display a crystalline structure with interplanar distances corresponding to CdS nanocrystals with polycrystalline wurtzite (hexagonal). The high structural and size definition of QDs is not commonly obtained in the biological synthesis of nanocrystals, where most nanostructures present high polydispersity and poorly defined structure models. These structural properties are in agreement with the spectroscopic properties of biosynthesized QDs.

In general, a drawback of CdS QDs biosynthesized by bacteria is the broad emission spectra that they present, a characteristic not desirable for fluorescence-based applications. The emission spectra of QDs produced by *Halobacillus* sp. DS2 after 1 and 3 h have full width at half maximum (FWHM) values of 85 and 110 nm, respectively. These values are higher than those obtained in QDs produced by some chemical methods (below 40 nm) but are lower than FWHM values reported for CdS QDs synthesized by bacteria. CdS QDs produced by *E. coli*, Antarctic *Pseudomonas* strains and acidophilic bacteria present FWHM values of 200, 215–225 and 220 nm, respectively^26,35^. The emission spectra of QDs biosynthesized by *Halobacillus* sp. DS2 are most probably consequence of the presence of organic or inorganic components on the surface of the nanocrystal. In this context, emission espectra of QDs produced by *Halobacillus* sp. DS2 are favored by the presence of NaCl during biosynthesis (Fig. S1). The unusual sharp maximum on the emission spectra of QDs biosynthesized in presence of NaCl was exacerbated at higher salt concentrations (4, 6 and 8%). Altogether, spectroscopic characteristics of biosynthesized QDs do not confirm the excitonic nature of nanoparticles and are probably consequence of trap-states on the QDs surface.

The chemical composition of biosynthesized QDs was determined by EDS and FT-IR. Obtained results indicate that CdS QDs are covered with an organic layer in which elements such as oxygen, carbon and phosphorus, probably come from proteins produced by *Halobacillus* sp. DS2 or from the cysteine molecule used to stimulate the production of H_2_S (Fig. 2). All these components have been previously described in other biological methods for QDs production^35^. The presence of chlorine is also expected in cells exposed to high concentrations of NaCl, mostly because it is a small anion of easy adsorption on reactive surfaces. The FTIR analysis of the organic composition of QDs indicates the presence of groups that most probably correspond to cysteine residues or protein molecules bound to the CdS nanocrystal through an S-Cd linkage (Fig. 7).

The HRXPS confirmed the presence of CdS and cysteine in the nanocrystals produced by halophilic bacteria. Interestingly, the presence of sodium and chlorine, mainly attributed to NaCl was also observed in QDs. NaCl presence is a novel characteristic of biosynthesized QDs, and is most probably consequence of NaCl interaction with biomolecules composing the organic capping of QDs like proteins or peptides.

Altogether, presented results indicate that the polyextremophyle bacterium *Halobacillus* sp. DS2 can synthesize CdS nanoparticles in presence of high NaCl concentrations in a process that is related, at least in part, to their capacity to generate S^2−^ in these conditions. Interestingly, QDs produced by halophilic bacteria display unique properties when compared with other biological QDs, such as narrow emission spectra (FWHM) and stability at high NaCl concentrations. The novel properties of these NPs are most probably consequence of the presence of NaCl and cysteine. Experiments to confirm this possibility are currently being developed in our lab.

Finally, the results of our work open new possibilities in the field of QDs biosynthesis and confirm the great potential of using microorganisms with the capacity to tolerate extreme conditions (osmolarity, acidity, temperature or metal stress, among many others) to synthesize nanoparticles with properties not easily found in nanomaterials produced by conventional methods (chemical or biological). Understanding the process of biosynthesis and determining the molecules involved will contribute to the development of new methods to produce nanomaterials with the specific properties that different industrial applications require.

## Methods

### Sample collection

Samples of soil, sediments and water were collected from different sites in the Atacama Salt Flat, Uyuni Salt Flat and the Dead Sea. Samples were stored at 4 °C until their processing.

### Isolation of bacterial strains

Samples (100 mg of soil or 100 μL of water) were suspended in 1 mL sterile distilled water (final volume) and vigorously stirred by vortexing (30 s). After that, 10 μL of each solution were used to inoculate 990 μL of LB culture medium and incubated at 28 °C. After 24 h, aliquots were seeded on LB agar plates and incubated at the same temperature for 24–48 h.

### Selection of salt-resistant bacteria

Resistant bacteria were selected by plating on LB agar plates supplemented with NaCl (8%). Bacterial growth at 28 °C was monitored after 24 and 48 h.

### Minimal Inhibitory Concentration (MIC)

MICs were determined using the protocol previously described by Elías *et al*.^65^. Solutions containing 5.5 mM CdCl_2_ were prepared in LB medium. Serial dilutions were set in 96-well microplates and inoculated with 5 μL of a previously grown bacterial culture. Microplates were incubated at 28 °C and bacterial growth was assessed after 24 h.

### Optimal growth temperature

Bacterial cultures were grown at 28 °C overnight and then diluted to an optic density at 600 nm (OD_600_) ~0.2. Serial dilutions were prepared and 5 μL of each dilution were used to inoculate LB + NaCl 8%-agar plates. Plates were incubated at 4, 15, 28 and 37 °C and bacterial growth was determined after 24 h.

### Bacterial growth in presence of NaCl

Bacterial cultures were grown at 28 °C overnight and then diluted to OD_600_~0.2. Serial dilutions were prepared and 5 μL of each dilution were used to inoculate LB-agar plates supplemented with 0, 2, 4, 8, 15 and 22% NaCl. Plates were incubated at 28 °C and bacterial growth was evaluated after 24 h.

### Effect of pH on bacterial growth

Bacterial cultures were grown overnight at 28 °C and then diluted to an OD_600_~0.2. 5 μL of each dilution were used to inoculate tubes with LB adjusted at several pHs (1–10). Tubes were incubated at 28 °C and bacterial growth was determined after 24 h.

### Sulfide detection assay

The generation of hydrogen sulfide in bacterial cultures was determined using lead acetate soaked papers as described by Shatalin *et al*.^66^. The assay was performed using 1 mL of bacterial cultures grown in 2 mL Eppendorf tubes to which a lead acetate paper was attached under the cap. Briefly, bacterial cultures were grown in 990 μL LB supplemented with several NaCl concentrations (0, 2, 4 and 8%) and grown at 28 °C until OD_600_~0.6. Then, 0.33 mM CdCl_2_ and 1 mM cysteine were added and the tubes were covered with a paper embedded in lead acetate (100 mM). Tubes were incubated at 28 °C and sulfide generation was determined after 6 h. Controls were performed in the absence of cysteine.

### Chemical synthesis of CdS nanoparticles

Chemical QDs were used as control for the assays of CdS stability at high NaCl concentrations. The synthesis at 1 h and 3 h of fluorescent nanoparticles was carried out at 90 °C using the protocol previously described by Pérez-Donoso *et al*.^17^.

### Intracellular biosynthesis of CdS nanoparticles

A bacterial pre-inoculum was used to inoculate (1:100) LB media supplemented with NaCl 0, 2, 4, 6 or 8% with or without 0.33 mM CdCl_2_. Then cultures were grown at 28 °C for 24 h. Samples were centrifuged 5 min at 10,000 *x g*, and the fluorescence of cell pellets and supernatants was evaluated after excitation at 360 nm using a short wave UV-transilluminator. *E. coli* was used as control.

### Extracellular biosynthesis of CdS nanoparticles

Bacteria were grown 24 h at 28 °C in LB media supplemented with NaCl 8%. Samples of 1 mL were then centrifuged 5 min at 10,000 *x g* and supernatants were discarded. Cells were suspended in buffer Tris-borax citrate 50 mM pH 8.0 amended with different NaCl concentrations (0, 2, 4, 6 and 8%). These solutions were supplemented with 2 mM cysteine in presence of 0.33 mM CdCl_2_. The fluorescence of supernatants was evaluated after excitation at 360 nm using a short wave UV-transilluminator as described before^26,27,32^.

### Purification of biosynthesized QDs

Extracellular QDs were purified from cell culture supernatants. Supernatants were filtrated using a 0.22 μm filter. Then, the nanoparticles were precipitated with a 1:1 (v/v) ethanol:water solution and mixed by vortexing^32^. Samples were incubated at 4 °C for 12 h, centrifuged 10 min at 10,000 *x g* and the supernatants discarded. Obtained NPs were dialyzed for 1 h with Tris-HCl pH 7.0 (100 mM), washed and concentrated using a 3 kDa filter (Millipore).

### Fluorescence of purified nanoparticles

Fluorescence and excitation spectra of purified nanoparticles were determined by using a multiplate reader, Synergy H1 (Biotek). Emission spectrum of purified nanoparticles was obtained after excitation at 360 nm. The excitation spectra of purified nanoparticles were obtained by measuring the fluorescence at 450 nm as described previously^27,32^.

The Quantum Yield of purified CdS QDs was determined. For this purpose, the fluorescence of 4 samples of QDs with different absorbances (between 0.01 and 0.1) was evaluated (excitation at 360 nm). Also, we use Nile Blue dissolved in ethanol (QY = 0.27) for this determination. Fluorescence spectra were measured to obtain the integrated fluorescence intensity (IFI, is defined as the area of the fluorescence spectrum) and this value was plotted versus the absorbance of the solution. The slope of both curves (*m*) and the refractive index of the solvents (*n*) (water: 1.333 and ethanol: 1.335) were used to calculate the QY of CdS QDs considering Nile blue as reference (R).The following equation was used^16,67^:

$$Q{Y}_{NPs}=Q{Y}_{R}[{m}_{NPs}/{m}_{R}]{[{{n}^{2}}_{NPs}/{{n}^{2}}_{R}]}^{-1}$$

### Transmission electron microscopy (TEM)

TEM measurements were performed using a FEI Tecnai G2 F20 S-Twin microscope, operated at 200 kV. A drop of the dispersed sample was left to dry out on a commercial carbon coated Cu TEM grid. TEM images were processed and analyzed with Digital Micrograph 3.9.0 (Gatan Inc) and The Gimp 2.4.0 software packages. A statistical study of TEM images was carried out to quantify nanoparticles sizes. This study consists of the size measurement (diameter) of about 200 particles per sample. The counts were then plotted as frequency histograms and the mean particle size was calculated. In addition, samples were chemically characterized by Energy-dispersive X-ray spectroscopy (EDX) and electron diffraction (ED).

### Fourier Transform Infrared Spectroscopy (FT-IR)

FT-IR was performed to purified QDs. Samples were dried using a vacuum centrifugal concentrator (IR Concentrator Micro-Cenvac NB-503CIR, N-Biotek Inc.) for 24 h. Then FT-IR spectra were determined using a Nicolet^TM^ iSTM10 FT-IR Spectrometer (Thermo Scientific Inc.) with a Smart iTRTM Attenuated Total Reflectance (ATR) accessory provided with a single bounce Ge crystal. The scan frequency was 4000 to 700 cm^−1^.

### X-ray photoelectron spectroscopy (XPS)

The chemical composition of biosynthesized NPs was studied by means of chemical binding energy of the elements through X-ray photoelectron spectroscopy technique (XPS). An XPS–Auger PerkinElmer spectrometer model PHI 1257 that includes an ultra-high vacuum chamber, a hemispheric electron energy analyzer and an X-ray source, with Kα radiation unfiltered from an Al (hν = 1486.6 eV) anode were used. The measurements were performed at 200 W using an emission angle of 70 to obtain information from the deep surface.

## Supplementary information

S1
