# Supplementary material for: Synthesis of salt-stable fluorescent nanoparticles (quantum dots) by polyextremophile halophilic bacteria
Source: Sci Rep. 2019 Feb 13;9:1953. doi: 10.1038/s41598-018-38330-8 (PMC6374371; doi:10.1038/s41598-018-38330-8)
Supplement: Supplementary file 1 — S1 [file 41598_2018_38330_MOESM1_ESM.pdf]

Synthesis of salt-stable fluorescent nanoparticles (quantum dots) by polyextremophile halophilic bacteria

Bruna N[1]., Collao B[1]., Tello A[1,2]., Caravantes P[1]., Díaz-Silva N[1]., Monrás JP[1]., Órdenes-Aenishanslins N[1]., Flores M[3]., Espinoza-Gonzalez R[3]., Bravo D[4] and Pérez-Donoso JM[1*]

1.- BioNanotechnology and Microbiology Lab, Center for Bioinformatics and Integrative Biology (CBIB), Universidad Andres Bello, Santiago, Chile.

2.- Laboratorio de Nanotecnología, Recursos Naturales y Sistemas Complejos, Facultad de Ciencias Naturales, Departamento de Química y Biología, Universidad de Atacama, Copiapó, Chile.

3.- Departamento de Ingeniería Química, Biotecnología y Materiales, Facultad de Ciencias Físicas y Matemáticas, Universidad de Chile, Santiago, Chile.

4.- Laboratorio de Microbiología Oral, Facultad de Odontología, Universidad de Chile, Santiago, Chile.

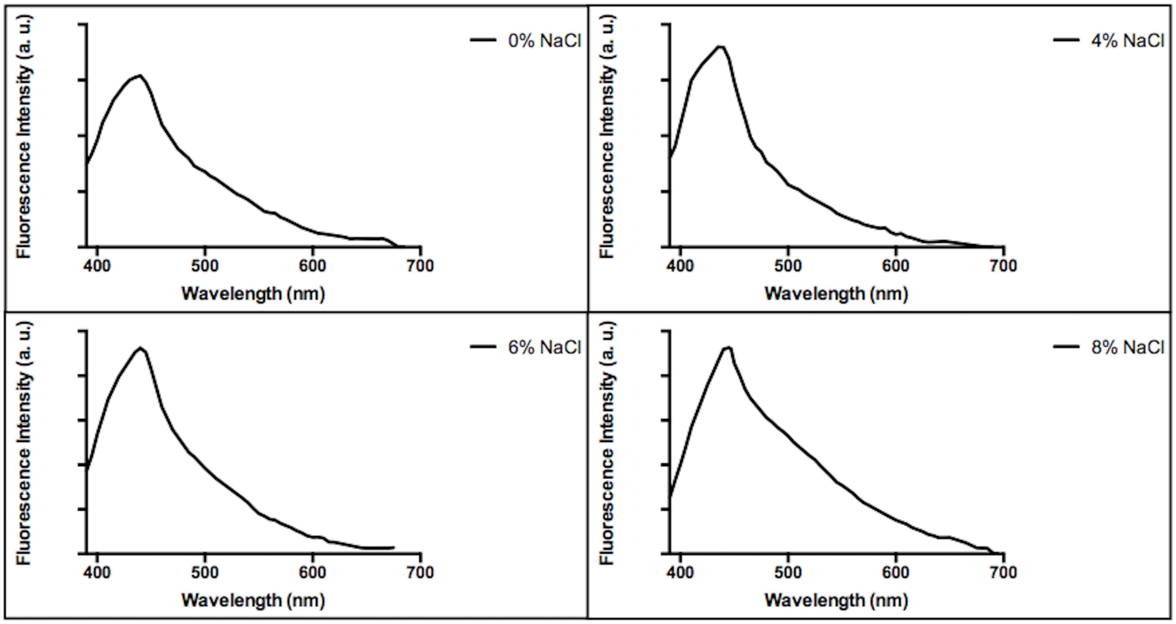

Figure S1. Emission spectra of QDs synthesized by *Halobacillus* sp. DS2 in presence of different NaCl concentrations (3 h biosynthesis).
